# Supplementary material for: Disorders in brassinosteroids signal transduction triggers the profound molecular alterations in the crown tissue of barley under drought
Source: PLoS One. 2025 Feb 3;20(2):e0318281. doi: 10.1371/journal.pone.0318281 (PMC11790124; doi:10.1371/journal.pone.0318281)
Supplement: S1 Table — The fragmentation parameters refer to the first Q3, which was used for quantification. (DOCX) [file pone.0318281.s001.docx]

Supplementary Table S1. Optimized HPLC/ESI-MS/MS parameters in multiple reaction mode (MRM) with positive ionization are listed for the brassinosteroids quantified in barley. The fragmentation parameters refer to the first Q3, which was used for quantification

| **Analyte** | **RT (min)** | **Q1** | **Q3** | **Q3** | **Fragmentation parameters** | | | |
| --- | --- | --- | --- | --- | --- | --- | --- | --- |
|  |  |  |  |  | **EP** | **DP** | **CE** | **CXP** |
| BL | 4.71 | 481.7 | 445.6 | 315.5 | 10 | 95 | 24 | 13 |
| d_3_ BL | 4.70 | 484.6 | 448.7 | 315.3 | 10 | 110 | 17 | 15 |
| CS | 4.91 | 465.7 | 429.5 | 299.5 | 10 | 90 | 25 | 15 |
| d_3_ CS | 4.90 | 468.7 | 432.6 | 299.3 | 10 | 90 | 24 | 13 |
| CT | 6.75 | 433.7 | 397.6 | 283.5 | 10 | 80 | 17 | 12 |
| d_3_ CT | 6.70 | 436.7 | 400.6 | 283.5 | 10 | 70 | 17 | 13 |

BL, brassinolide; CS, castasterone; CT, cathasterone; EP, entrance potential; DP, declustering potential; CE, collision energy; CXP, collision cell exit potential.
