# Supplementary figures and images for: Disorders in brassinosteroids signal transduction triggers the profound molecular alterations in the crown tissue of barley under drought
Source: PLoS One. 2025 Feb 3;20(2):e0318281. doi: 10.1371/journal.pone.0318281 (PMC11790124; doi:10.1371/journal.pone.0318281)

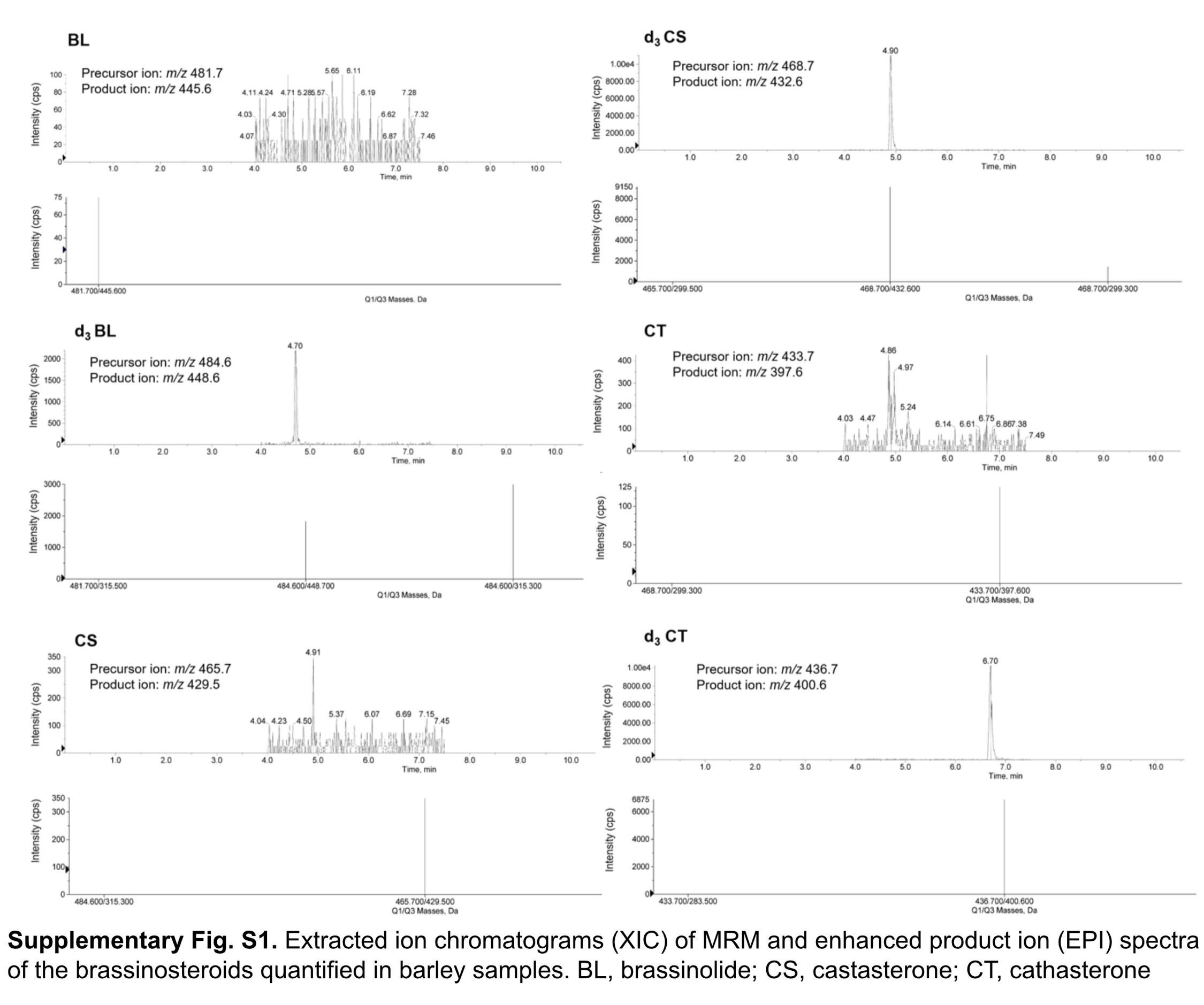

Supplement: S1 Fig — BL, brassinolide; CS, castasterone; CT, cathasterone. (TIF) [file pone.0318281.s009.tif]

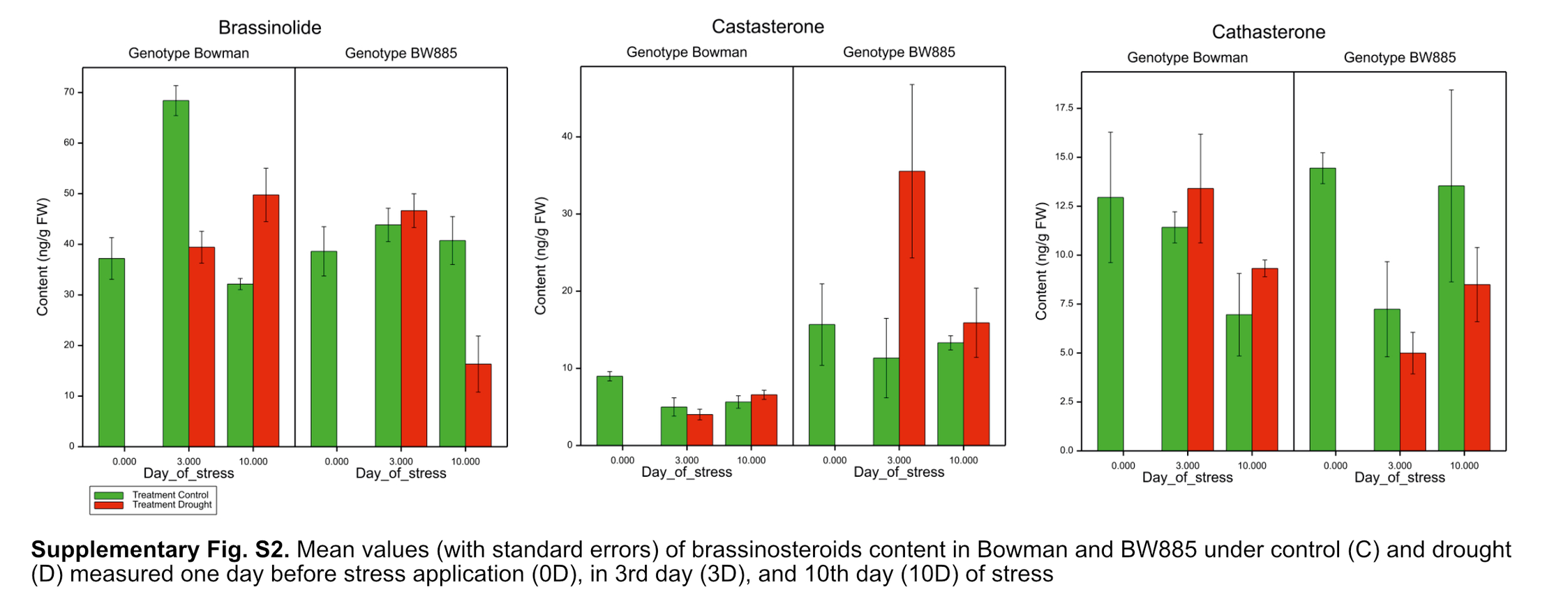

Supplement: S2 Fig — Mean values (with standard errors) of brassinosteroids content in Bowman and BW885 under control (C) and drought (D) measured one day before stress application (0D), in 3rd day (3D), and 10th day (10D) of stress. (TIF) [file pone.0318281.s010.tif]

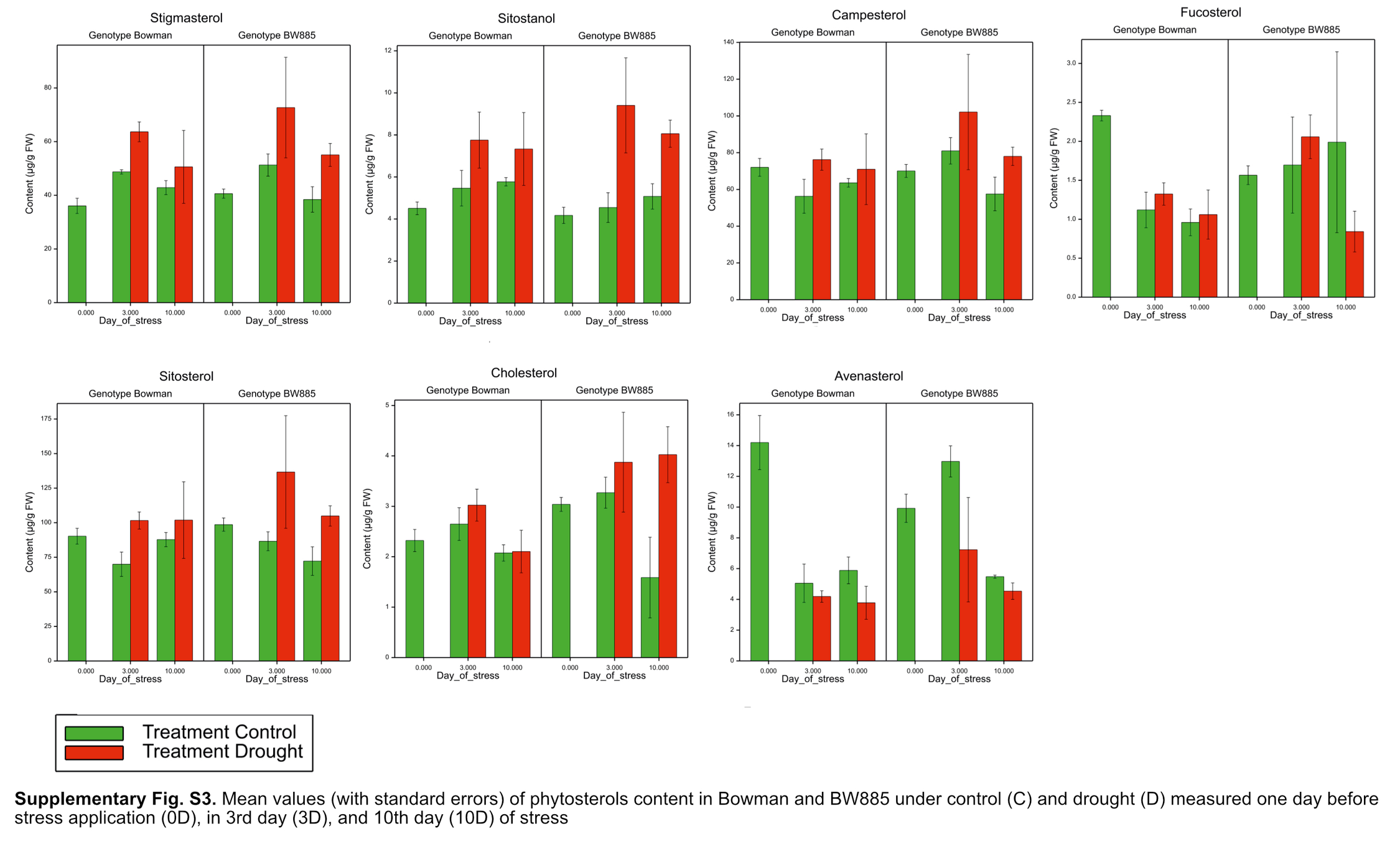

Supplement: S3 Fig — Mean values (with standard errors) of phytosterols content in Bowman and BW885 under control (C) and drought (D) measured one day before stress application (0D), in 3rd day (3D), and 10th day (10D) of stress. (TIF) [file pone.0318281.s011.tif]
