# Supplementary material for: Disorders in brassinosteroids signal transduction triggers the profound molecular alterations in the crown tissue of barley under drought
Source: PLoS One. 2025 Feb 3;20(2):e0318281. doi: 10.1371/journal.pone.0318281 (PMC11790124; doi:10.1371/journal.pone.0318281)
Supplement: S4 Fig — Mean values (with standard errors) of phenotypic traits observed for Bowman and BW885 under control (C) and drought (D); (B) principal component biplot for analyzed traits (T1-T22): green circle, BW_C; red circle, BW_D; green triangle, BW885_C; red triangle, BW885_D. (PDF) [file pone.0318281.s012.pdf]

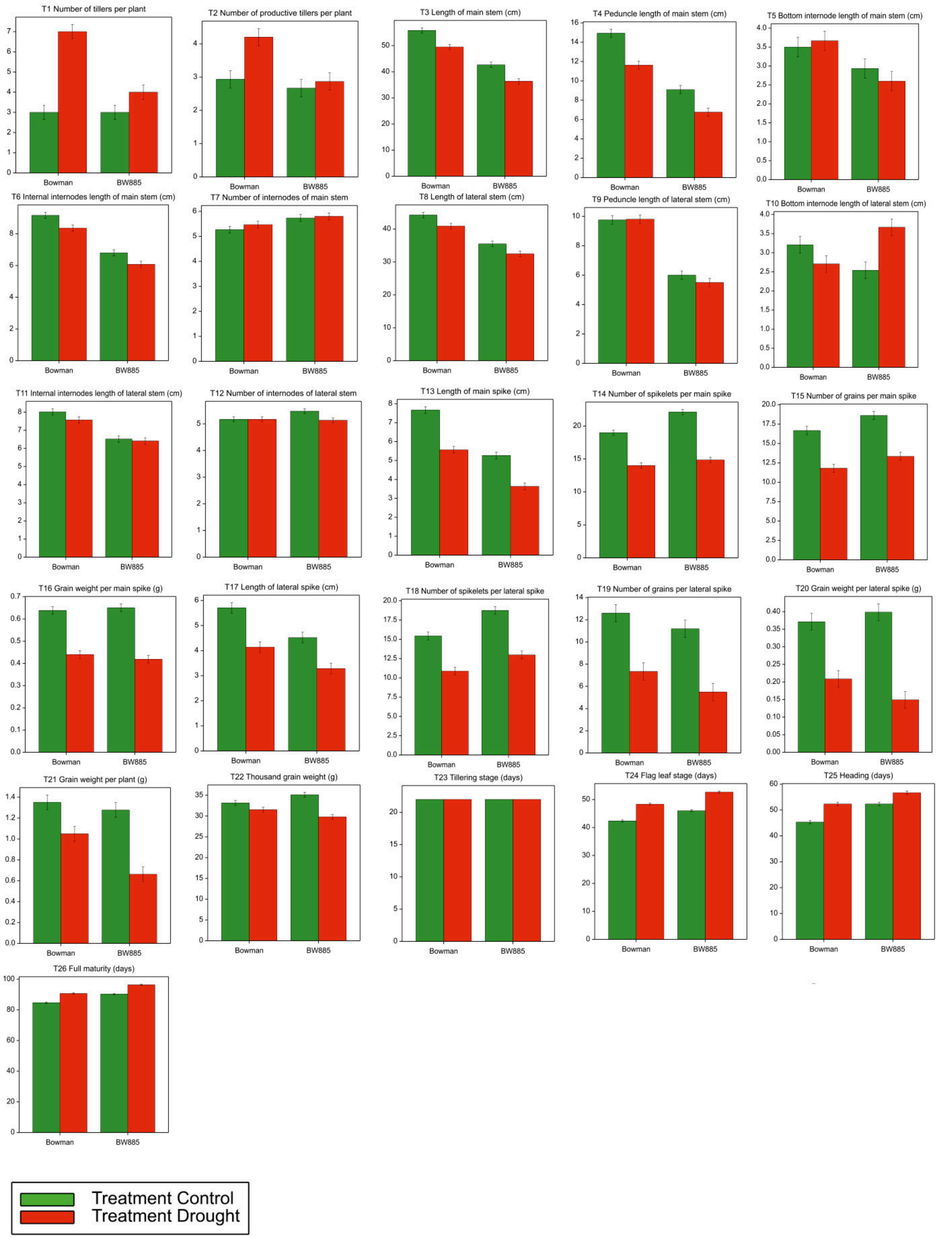

Supplementary Fig. S4A. Mean values (with standard errors) of phenotypic traits observed for Bowmann and BW885 under control (C) and drought (D)

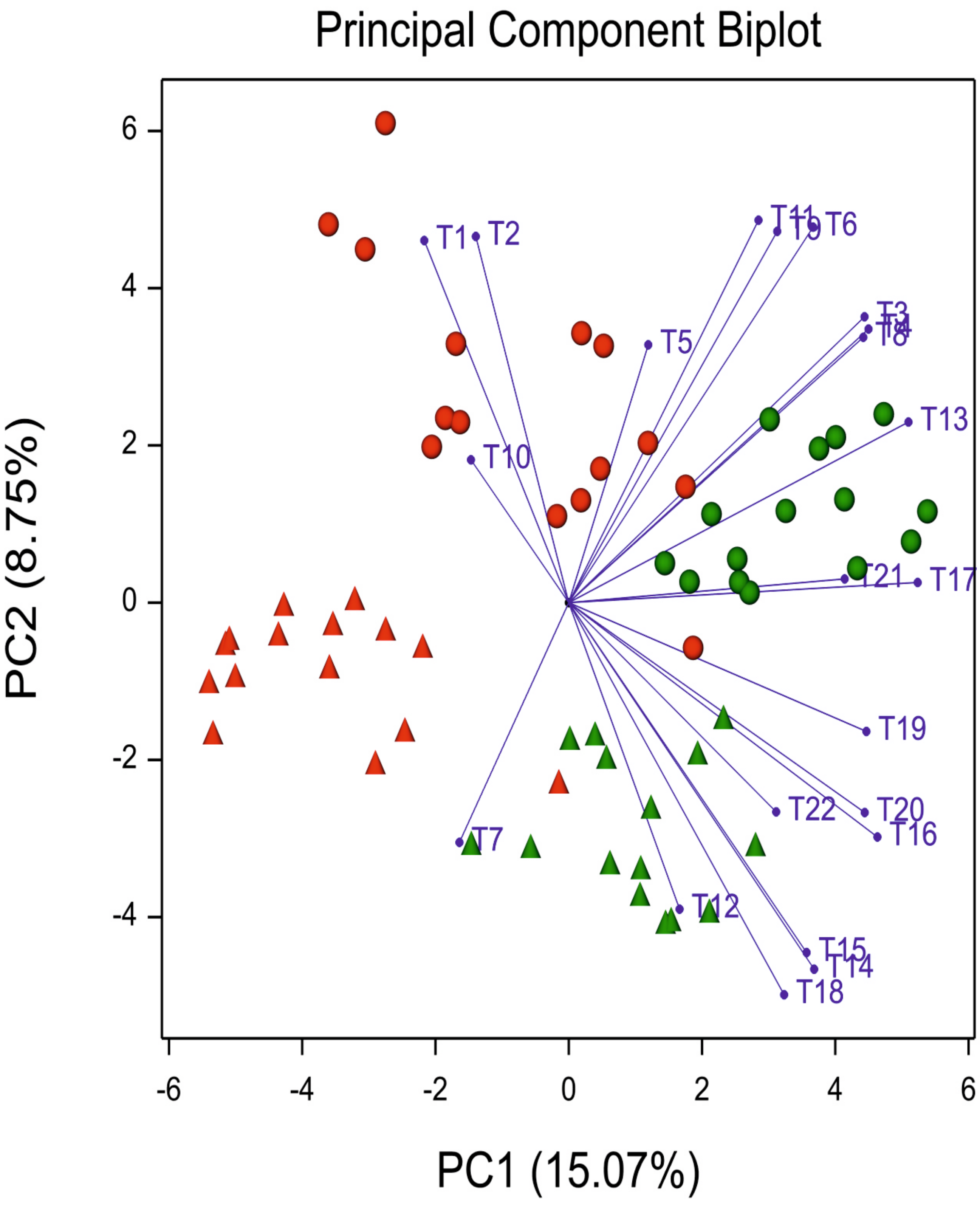

Supplementary Fig. S4B. Principal component biplot for analyzed traits (T1-T22): green circle, Bowman in control; red circle, Bowman in drought; green triangle, BW885 in control; red triangle, BW885 in drought
