# Supplementary material for: Disorders in brassinosteroids signal transduction triggers the profound molecular alterations in the crown tissue of barley under drought
Source: PLoS One. 2025 Feb 3;20(2):e0318281. doi: 10.1371/journal.pone.0318281 (PMC11790124; doi:10.1371/journal.pone.0318281)
Supplement: Attachment — Submitted filename: Response to Reviewers.docx [file pone.0318281.s014.docx]

Dear Reviewer,

To address your comment regarding the methodology for phytohormone determination, we have significantly expanded the description in the Materials and Methods section. Additionally, we have introduced a supplementary table presenting the optimized MRM parameters, and, as per your suggestion, we have included a supplementary figure featuring the extracted ion chromatograms (XIC) of MRM and enhanced product ion (EPI) spectra of the brassinosteroids analyzed in the barley samples. These revisions have resulted in a renumbering of the supplementary materials. The citation Dias et al. 2018 has been removed that resulted in renumbering of cited literature within the manuscript.

*Did you made sure that matrix effects, when working with 100 mg of plant material without using SPE or derivatization, is not disturbing your quantification?*

Response: Matrix effects are certainly reducing a little bit the sensitivity of the MS/MS but it is not disturbing the quantification. Peaks were in some cases small but clear enough to allow a proper quantification (see newly submitted suppl. file).

Many thanks for all your comments and criticisms raised, which have served to improve our work. We hope these changes comprehensively address your concerns and enhance the clarity and robustness of the manuscript.
